# Identification of distinct genotypes in circulating RSV A strains based on variants in the virus replication-associated genes

**DOI:** 10.1128/jvi.00990-24

**Published:** 2024-07-15

**Authors:** Abdulafiz O. Musa, Sydney R. Faber, Kaitlyn Forrest, Kenneth P. Smith, Shaon Sengupta, Carolina B. López

**Affiliations:** 1 Department of Molecular Microbiology, Washington University School of Medicine, Saint Louis, Missouri, USA; 2 Center for Women's Infectious Diseases Research, Washington University School of Medicine, Saint Louis, Missouri, USA; 3 Department of Pediatrics, Perelman School of Medicine, University of Pennsylvania, Philadelphia, Pennsylvania, USA; 4 Division of Neonatology, Children’s Hospital of Philadelphia, Philadelphia, Pennsylvania, USA; 5 Department of Pathology and Laboratory Medicine, Perelman School of Medicine, University of Pennsylvania, Philadelphia, Pennsylvania, USA; 6 Infectious Disease Diagnostics Laboratory, Children’s Hospital of Philadelphia, Philadelphia, Pennsylvania, USA; The Ohio State University, Columbus, Ohio, USA

**Keywords:** respiratory syncytial virus, genotypes, polymerase L, replication-associated genes

## Abstract

Respiratory syncytial virus (RSV) is a common cause of respiratory infection that often leads to hospitalization of infected younger children and older adults. RSV is classified into two strains, A and B, each with several subgroups or genotypes. One issue with the definition of these subgroups is the lack of a unified method of identification or genotyping. We propose that genotyping strategies based on the genes coding for replication-associated proteins could provide critical information on the replication capacity of the distinct subgroups, while clearly distinguishing genotypes. Here, we analyzed the virus replication-associated genes N, P, M2, and L from *de novo* assembled RSV A sequences obtained from 31 newly sequenced samples from hospitalized patients in Philadelphia and 78 additional publicly available sequences from different geographic locations within the United States. In-depth analysis and annotation of variants in the replication-associated proteins identified the polymerase protein L as a robust target for genotyping RSV subgroups. Importantly, our analysis revealed non-synonymous variations in L that were consistently accompanied by conserved changes in its co-factor P or the M2-2 protein, suggesting associations and interactions between specific domains of these proteins. Similar associations were seen among sequences of the related human metapneumovirus. These results highlight L as an alternative to other RSV genotyping targets and demonstrate the value of in-depth analyses and annotations of RSV sequences as it can serve as a foundation for subsequent *in vitro* and clinical studies on the efficiency of the polymerase and fitness of different virus isolates.

**IMPORTANCE:**

Given the historical heterogeneity of respiratory syncytial virus (RSV) and the disease it causes, there is a need to understand the properties of the circulating RSV strains each season. This information would benefit from an informative and consensus method of genotyping the virus. Here, we carried out a variant analysis that shows a pattern of specific variations among the replication-associated genes of RSV A across different seasons. Interestingly, these variation patterns, which were also seen in human metapneumovirus sequences, point to previously defined interactions of domains within these genes, suggesting co-variation in the replication-associated genes. Our results also suggest a genotyping strategy that can prove to be particularly important in understanding the genotype-phenotype correlation in the era of RSV vaccination, where selective pressure on the virus to evolve is anticipated. More importantly, the categorization of pneumoviruses based on these patterns may be of prognostic value.

## INTRODUCTION

Respiratory syncytial virus (RSV) is a negative-sense single-stranded RNA virus that belongs to the family *Pneumoviridae* and order Mononegavirales. RSV circulates seasonally around the world, and it is estimated to infect every child at least once before the age of three, with the possibility of reinfections throughout life (1, 2). RSV leads to a wide variety of clinical outcomes that range from a mild cold to bronchiolitis, pneumonia, and death (2). Each year, in the United States (US) alone, RSV leads to an estimated 58,000–80,000 hospitalizations in children under the age of 5 (3, 4). RSV is also a major cause of morbidity in immunocompromised individuals and elderly adults (5, 6). Recent advances in RSV antivirals and vaccines for selected populations are encouraging (7–12). However, given the circulating nature of RSV among the human population, the abundance of infections, and the wide variety of clinical manifestations, there is a need for a better understanding of the genomic determinants of RSV pathogenesis. A key step in this direction is to improve the identification of RSV genotypes that are associated with different degrees of virus replication and pathogenicity.

The RSV genome is composed of 10 genes: NS1, NS2, N, P, M, SH, G, F, M2, and L. These genes encode 11 proteins as the M2 gene contains two overlapping open-reading frames that code for two proteins, M2-1 and M2-2 (13). The initial stage of RSV infection is determined by the virus surface glycoproteins G and F that mediate the binding of the virus to its receptor and fusion with the host cell membrane, respectively (14). The G protein is subjected to high selective pressure that causes it to frequently mutate (15). The F protein, also found on the surface of the virus, is fairly conserved among different RSV strains (14, 15). Once the virus enters a cell, viral transcription and genome replication are mediated by the polymerase L and its co-factors N, P, and M2-1 (13, 16). Additionally, M2-2 has been shown to play a role in the switch between transcription and replication, suggesting a role with the ribonucleocapsid complex (17–19).

Since its first discovery in 1956 (20, 21), RSV has been classified into two main serotypes, RSV-A and RSV-B (22, 23), both of which can be further divided into multiple genotypes. Historically, due to its relatively smaller size and high variability, the evolutionary events in the G gene have been used to genotype RSV. In most cases, RSV genotypes can be distinctly defined based on the second hypervariable region (HVR2) or ectodomain of G (24–29). Because G variations may impact receptor binding, G-based genotyping is a good method to identify variants with different entry abilities. Whole-genome sequencing has more recently been reported as an alternative method for genotyping, and it has been shown to better represent RSV genotypes in the population (30–33). M2-2 sequencing has also been proposed as an alternative genotyping method, as this is one of the smallest viral proteins with a low level of conservation (34). Notably, no current routine genotyping method focuses on the viral polymerase and its associated genes despite the critical role of these proteins in viral replication and infection.

The lack of consensus on how to determine RSV genotypes makes it challenging to track circulating strains among the various reported sequences and even more difficult to predict associations with the replicative capacity of the different RSV variants. To assess whether variations in the replication-associated proteins can identify RSV genotypes, we took an in-depth look at the variants present in *de novo* assembled whole-length RSV genomes circulating in the US between 2012-2023. We included 31 newly sequenced samples obtained from a cohort of hospitalized children in Philadelphia, as well as 78 publicly available data sets of RSV A sequences obtained from samples around the country. Comparing RSV A sequences from samples in different US regions between the years 2012 and 2023, we found predictable RSV A genotypes distinguished solely by variants in the L gene. Remarkably, non-synonymous variations in L were consistently accompanied by conserved changes in the L co-factors P or M2-2, suggesting the co-variation of replication-association proteins in circulating genotypes. Furthermore, these non-synonymous variations accurately predicted RSV genotypes based on the whole-genome sequence. This report demonstrates the importance of in-depth analysis of full-length viral sequences to uncover potentially important functional changes in viral proteins, and it identifies clusters of associated variations in polymerase-related genes that can be used to genotype RSV with high accuracy.

## RESULTS

### The G and L genes have the highest number of variations among RSV A variants circulating in the United States during 2012– 2023

**Fig 1:**
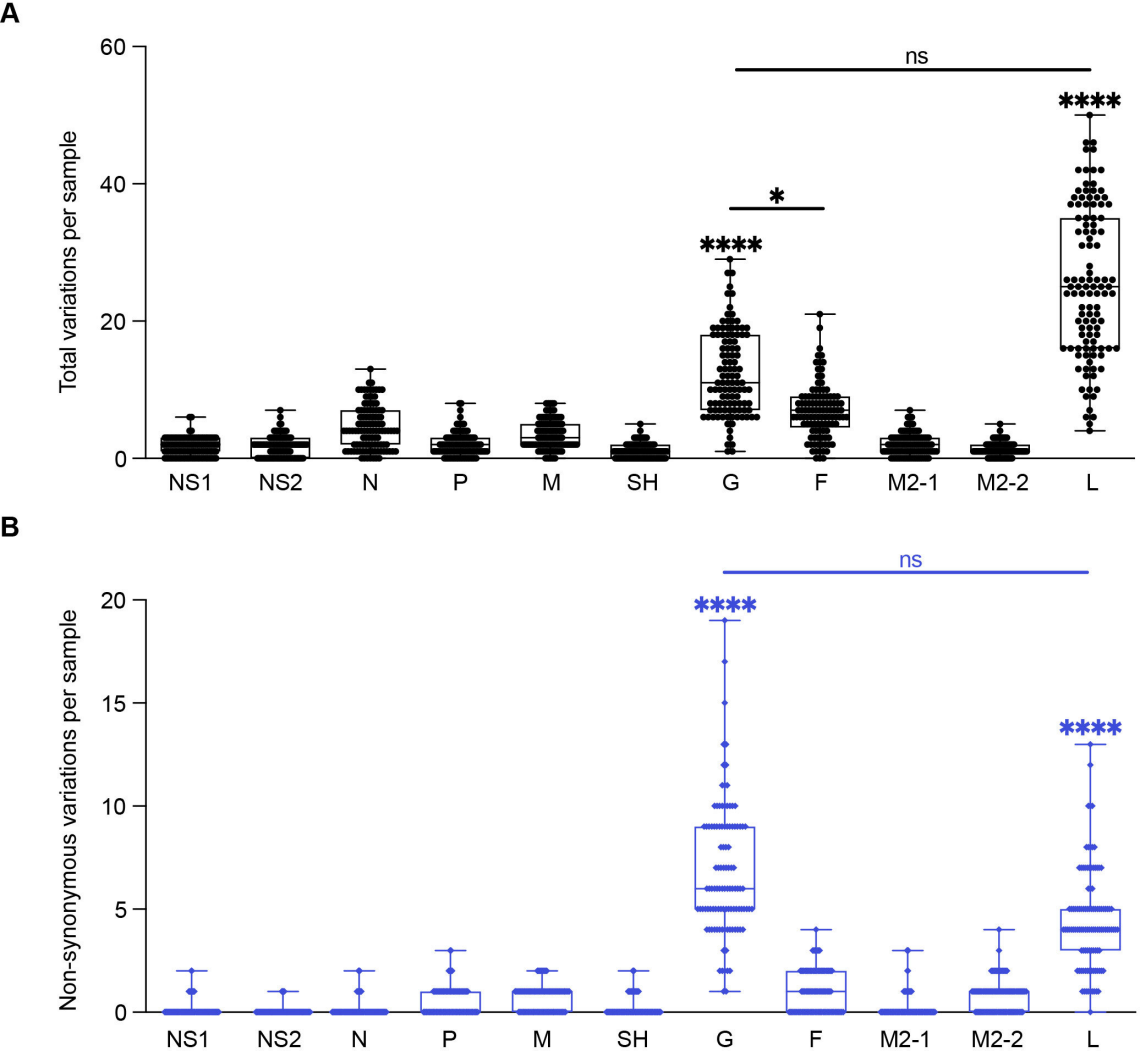
Summary of genetic variations of RSV A 11 CDS regions. Each point represents the number of variants per sample, *N* = 109. (**A**) Total variations per sample for each CDS region. (**B**) Non-synonymous variations per sample for each CDS region. One-way analysis of variance (ANOVA) non-parametric Kruskal-Wallis tests were performed for statistical significance between CDS regions. ns *P* > 0.1234; *P > 0.03; ****P < 0.0001. Comparisons between all the CDS regions to G or L were **** unless otherwise noted by a line between CDS regions.

To identify the genes with the highest number of variations in recently circulating RSV A variants, we initially sequenced and *de novo* assembled full-length genomes of 31 samples obtained from pediatric patients at the Children’s Hospital of Philadelphia (CHOP). We completely annotated these sequences and submitted them under GenBank: PP525(296–326). Using the consensus sequence of all the samples as a reference to identify variants between the different samples, we observed the highest number of total and non-synonymous variations in the coding regions of G and L. The M2-2 coding region showed the highest ratio of non-synonymous to total variation, and NS2 was the most conserved (Table S1). We expanded our data set with 78 additional RSV A sequences from the National Center of Biotechnology Information (NCBI) database that accounted for different locations in the United States as well as a broader range of years the sequences were collected (Table 1; Table S2). In the combined cohort of 109 RSV A full-length sequences, we again observed the most total and non-synonymous variations in the G and L genes with the M2-2 showing the highest ratio of non-synonymous to total variation (Table 2; Fig. 1).

**TABLE 1:** Distribution of the 109 sequences used in this study including the years samples were collected and their locations by state^*a*^

Year	US location								Total
	AR	AZ	MA	OR	PA	TN	WA	UNKN	
2012				1	3	2		2	8
2013					7			1	8
2014				2	6			2	10
2015			1		3		4	3	11
2016			3		9		5	3	20
2017		1			1			2	4
2018					1		1	2	4
2019	1	2						7	10
2020	2	2			1			5	10
2021							1	3	4
2022	1	1				1	7		10
2023	1	3					5	1	10
Total	5	9	4	3	31	3	23	31	109

^*a*^ All 31 PA samples are from the CHOP B Cohort and other sequences were randomly selected from NCBI. UNKN indicates that sequences are of unknown origin within the US.

**TABLE 2:** Computed variations for all 109 samples showing the combined number of substitutions, insertions, and deletions per coding region^*a*^

Gene	Total number of variations	Number of non-synonymous variations	Non-synonymous/ total variations
NS1	178	15	0.084
NS2	190	6	0.032
N	493	15	0.030
P	220	56	0.255
M	376	73	0.194
SH	120	24	0.200
G	1,348	743	0.551
F	749	107	0.143
M2-1	219	27	0.123
M2-2	145	86	0.593
L	2,776	486	0.175

^*a*^ Total variations were deduced from the nucleotide sequence alignment, and non-synonymous variations were deduced from the amino acid alignment.

### Non-synonymous variations in the L gene associate with specific variations in other replication-related genes that can predict genotypic clusters

We next took a closer look at the non-synonymous variations across the coding regions of the replication-related genes (N, P, M2, and L) in the 109 sequences, and annotated the non-synonymous variations for each sample compared against the consensus sequence (Table S3). We found associations of variations between L and one or more of the other replication genes. We marked out the similar variations in each coding sequence (CDS) region that appeared more than two times and categorized the sequences into groups named R1–R6 based on the observed associations (Table 3; Table S4). Each group contained a varying number of sequences independent of year or location (Table S5).

**TABLE 3:** Definition of groups based on repetitive non-synonymous variations often seen in combination across replication-associated genes^*a*^

Group name	Variations in P	Variations in M2-1	Variations in M2-2	Variations in L
R1	I66T/ P34L		N46S	G1725E/ H598Y/ D1731G/ V335I/ N215S
R2	T69I		C26Y	S1723G/ A1927T/ V1943D/ I139T/ T179A
R3		N174S/T180A/S182G	L37P	N143D/ T179S/ I1653V/K1661N/ S100L/ L953M/S1593N/ S1789F/ H1707Q/A2014/ Y2163N
R4	T92M		T79A	L835M/ A1537S/ N547S/P1720L/ V1943I
R5	N59S			R511K/ R1759K
R6				No variations

^*a*^ Variations included were observed in more than two sequences, and the groups shown account for all 109 RSV A sequences. N is not included here as there was no significant recurrence of non-synonymous variations observed. Within each CDS, a slash “/” indicates another variation seen in the group. For detailed associations, see Table S4.

We separated the sequences in each group based on the variation patterns observed (Table 4). R1 group contained a diverse set of variations. However, most of the R1 sequences contained L G1725E which associated 44% of the time with M2-2 N46S. Within the sequences containing L G1725E, three sequences had an additional L variation: N215S, which associated 100% of the time with variants P P34L and M2-2 N46S. Sequences with an additional L variation, V335I, associated 50% of the time with the P variant I66T. The variant patterns in the R2 and R4 groups were more consistent as at least 75% of the sequences had clear associations of variations in P, M2-2, and L. Interestingly, the R3 group essentially contained L: N143D, T179S, I1653V, K1661N of which 78% did not associate with any variants in P, M2-1, or M2-2. The remaining percentage of the group did, however, associate with either M2-2 L37P (13%) or a trio of M2-1 variants: N174S, T180A, S182G (9%). Out of the entire cohort analyzed, R3 was the only group to consistently contain variants in M2-1. Although the R5 group had a limited number of sequences, all the sequences in this group were composed of the variants P N59S and L R511K and R1759K.

**TABLE 4:** Detailed variation patterns observed in each group^*a*^

Group name	Variations in P	Variations in M2-1	Variations in M2-2	Variations in L	Number of sequences
R1				G1725E	12
			N46S	G1725E	7
			N46S	H598Y, G1725E, D1731G	4
	P34L		N46S	N215S, G1725E	3
			N46S		3
				H597Y, G1725E	3
			N46S	H598Y, D1731G	2
				V335I, G1725E	2
				H598Y	2
	I66T			V335I, G1725E	2
	I66T			V335I	1
			N46S	H598Y, G1725E	1
				V335I	1
R2	T69I		C26Y	S1723G, A1927T, V1943D	9
	T69I		C26Y	I139T, S1723G, A1927T, V1943D	3
	T69I		C26Y	T179A, S1723G, A1927T, V1943D	1
	T69I		C26Y	S1723G, V1943D	1
				S1723G, V1943D	1
				T179A, S1723G, V1943D	1
				T179A	1
R3				N143D, T179S, I1653V, K1661N	12
			L37P	N143D, T179S, I1653V, K1661N	4
				N143D, T179S, S1593N, I1653V, K1661N, S1789F, Y2163N	4
		N174S, T180A, S182G		N143D, T179S, I1653V, K1661N	3
				S100L, N143D, T179S, L935M, I1653V, K1661N	3
				N143D, T179S, I1653V, K1661N, A2014T, Y2163N	2
				S100L, N143D, T179S, L935M, I1653V, K1661N, H1707Q	2
				N143D, T179S, I1653V, K1661N, A2014T	1
				S100L, T179S, L935M, I1653V, K1661N, H1707Q	1
R4	T92M		T79A	L835M	6
	T92M		T79A	A173S, L835M	3
	T92M			N547S, L835M, P1720L, V1941I	3
				
R5	N59S			R511K, R1759K	3
R6					2

^*a*^ N is not included here as no significant recurrence of non-synonymous variations were observed.

**Fig 2:**
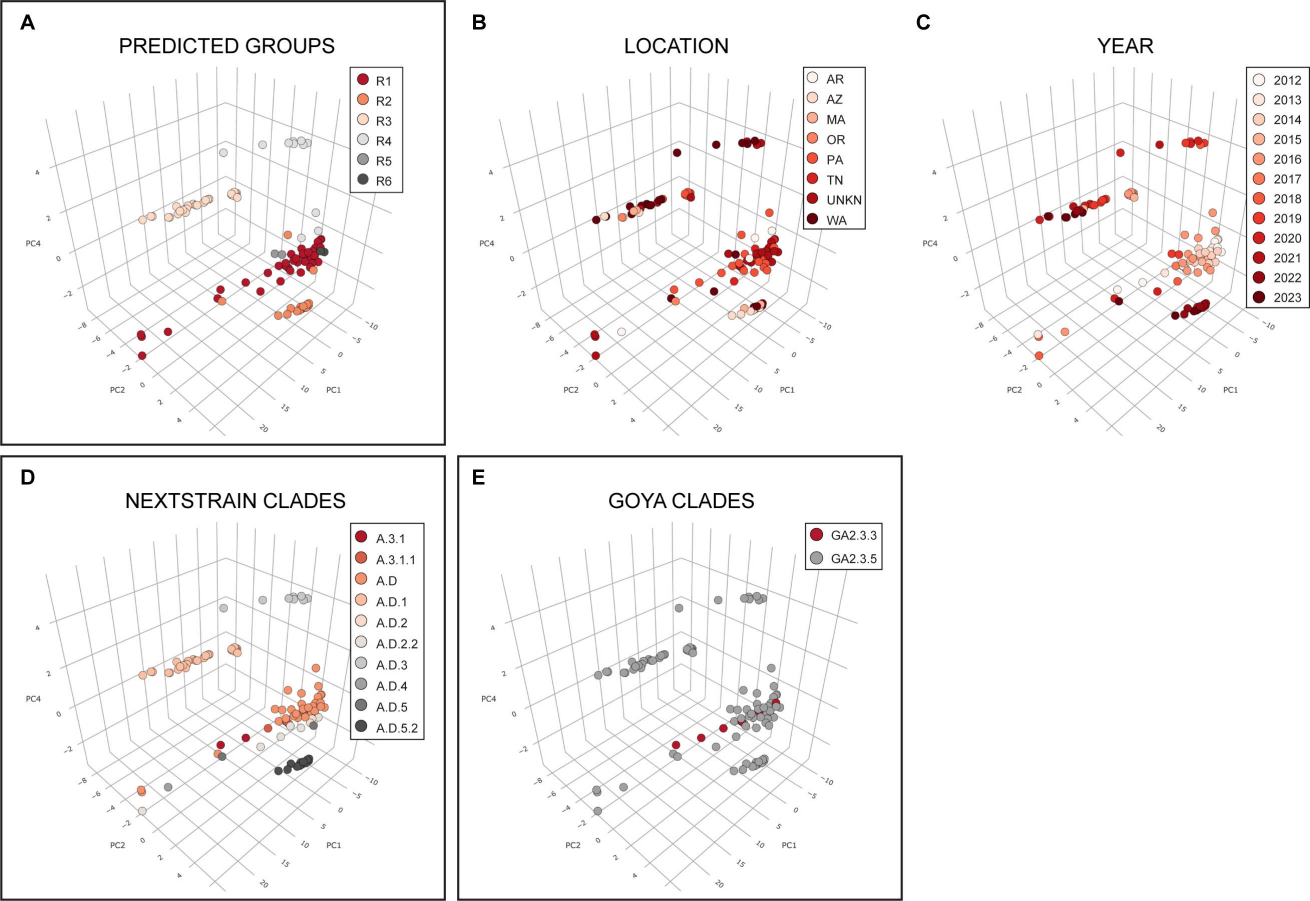
Principal component analysis (PCA) of the 109 RSV A sequences analyzed in this study. Plots outlined in squares are shown as a comparison between our predicted groups and other genotyping methods. Data points (circles) in each plot were labeled based on (**A**) predicted groups, (**B**) location in the United States, (**C**) year the sample was collected, (**D**) Nextstrain clades classification, and (**E**) Goya clades classification. Each data point represents a sequence. The distance between two data points on the plot depicts the similarity between the sequences. Hence, a cluster of data points means that those sequences are of similar clade phylogenetically. The percentage of variance in all the plots are PC1 = 58.71%, PC2 = 21.78%, and PC4 = 2.52%. Abbreviations in the “LOCATION” legend indicate the state the sequence was collected from. UNKN is used when a US state was not assigned to the sequence.

Next, we generated a principal component analysis (PCA) plot with the uncorrected pairwise distances of the 109 full-length sequences and labeled them with our predicted genotypic groups R1–R6 (Fig. 2A). We observed that most of the sequences distinctly clustered together based on the pre-determined groups, which supports the predicted associations among related sequences. Additionally, we labeled each sequence by the region or the year the sequences were obtained and found them to be independent of the associations (Fig. 2B and C).

We also employed the Nextclade web tool to assign genotypes and depict the phylogeny of the 109 samples based on two recently proposed classifications – Nextstrain clades and Goya clades. According to the documentation, a new Nextstrain clade is determined when a strain has at least two unique mutations in the whole genome different from the parental strains with other phylogenetic support. On the other hand, the Goya clade employs the statistical significance of the p-distance and bootstrap values in the phylogenetic tree to redefine the traditional “GA” nomenclature commonly used for RSV sequences. This method also identified that the G-ectodomain is the minimum genomic region that should be analyzed to provide reliable RSV genotypes (33, 35, 36).

**Fig 3:**
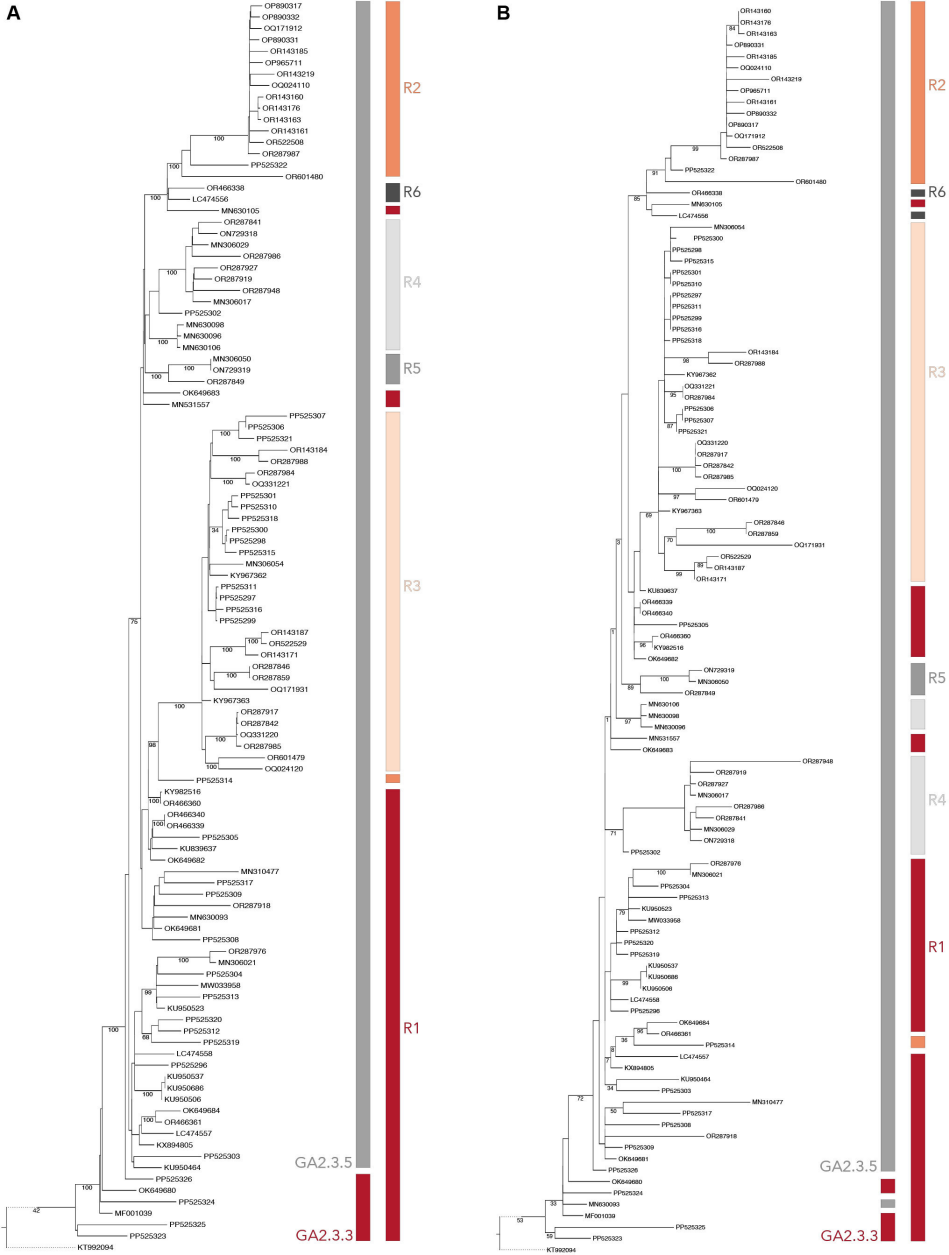
Maximum likelihood (ML) phylogeny tree of 109 RSV A (**A**) full-length sequences and (**B**) CDS region of G. Color bars represent assigned genotypes based on the GA clades and our predicted groups (R1–R6). The trees were rooted on reference sequence—KT992094.1. Dotted lines were used to shorten the distance between the reference and the rest of the sequence for easier visualization.

The Nextstrain clades classification resulted in 10 clades and the Goya clade classification resulted in only two clades, GA2.3.3 and GA2.3.5 (Table S6). We labeled the previously shown PCA plot (Fig. 2A) with both classification assignments from Nextclade (Fig. 2D and E). Even though there are limitations to the number of clusters that can be visualized on the PCA, we found the Nextstrain clade classification to be similar to our predicted groups. Furthermore, we supported our results by showing a comparison of the maximum-likelihood phylogenetic trees based on full-length and G CDS region sequences annotated with our R predicted groups and the GA clades (Fig. 3). The phylogenetic trees show that the clades formed using the full-length sequence and the G are similar. The assigned R groups reasonably matched the organization of the clades observed in the full-length tree. The traditional GA clade assignment on both trees underrepresented the genotypes for the sequences. These results confirm that the non-synonymous variations in the replication-associated genes can be used to identify distinct genotypes among RSV sequences.

We were curious if these associations could be applied to the closely related human metapneumovirus (HMPV). Therefore, we analyzed 21 HMPV A full-length sequences in the US using the reference sequence NC_039199.1 (GenBank) as a guide to observe variations patterns within the replication-associated genes. Our results in Table 5 and Table S7 show that, similar to RSV, there are specific associations among replication-associated genes in HMPV. Moreover, we were able to predict their genotype groups based on these associations. We named these groups M1, M2, and M3. Additional analyses are required to compare how these predicted groups relate to other HMPV genotyping methods. However, the distinct associations observed clearly define the clades visualized in the phylogenetic tree (Fig. S1).

**TABLE 5:** Definition of groups based on repetitive non-synonymous variations often seen in combination across replication-associated genes of 21 HMPV A sequences in the United States^*a*^

Group name	Variations in N	Variations in P	Variations in M2-1	Variations in M2-2	Variations in L
M1	Y220H/ A92T/V	R241K/ S44N/ R79K	T183I	S49F	G108S/ G132S/ R530K/ D760G/ T954A/ I1481V/ R1916K/ R898K/ T429N/ V79I/ V331A/T
M2		L277P/ I280L/ E282D			T505A/ M1609I/ I772V/ V1107I
M3		L277I	L118I		

Group name	Number of sequences	Range of years	US locations
M1	9	2004–2016	AR, NM, OH
M2	6	2015–2019	WA, NM
M3	6	2016	NM

^*a*^ Variations included were observed in more than two sequences. The lower section of the table shows the distribution of the predicted groups by year and location in the United States by states.

### Non-synonymous variations in replication-associated genes concentrate in protein-protein interaction domains

**Fig 4:**
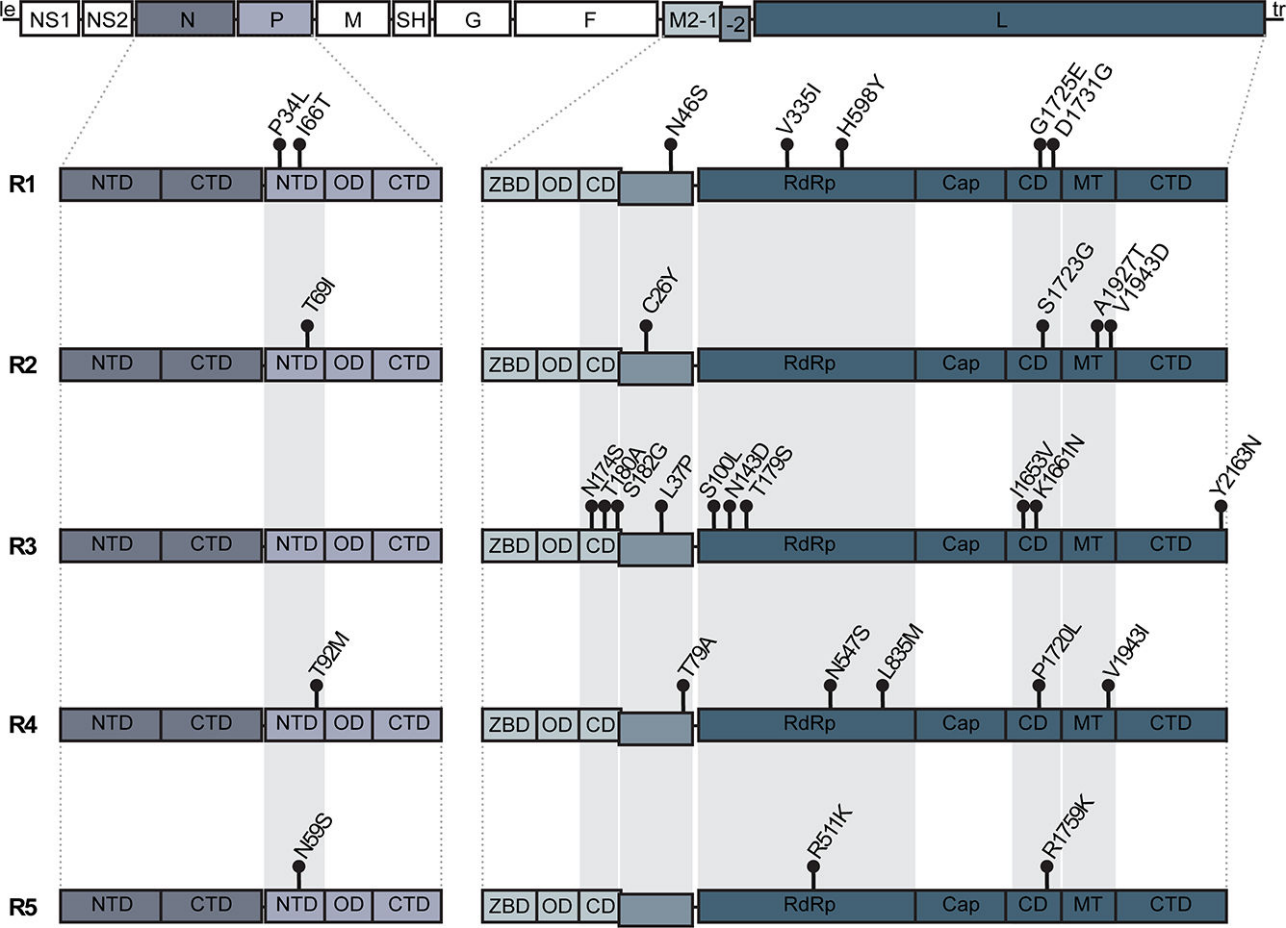
Annotation of non-synonymous variations observed within the domains of RSV A replication-associated genes for each predicted R group. The top illustration is the full-length genome of RSV with colored regions depicting the replication-associated genes. The illustrations below are zoomed in on the domains of the replication-associated genes. All variations shown in each predicted group, i.e., R1–R5 were seen at least three times along with other variations within the same predicted group. Areas shaded gray along R1–R5 indicate consistent domains with variations. R6 was omitted as it had no variation present in any of the domains. Abbreviation key as follows: NTD, N-terminal domain; CTD, C-terminal domain; OD, oligomerization domain; ZBD, zinc-binding domain; M2-1 CD, core domain; RdRP, RNA-dependent RNA polymerase; Cap, cap addition domain; L CD, connector domain; MT, cap methylation domain.

To determine if specific domains of the RSV replication-associated proteins were susceptible to variation, we annotated the variants observed in the different groups to their respective predicted domain residues (Fig. 4) (16). All the variants in P were found in the N-terminal domain (NTD) which is thought to bind to M2-1, specifically regions of the core domain, as well as interact with RNA-free N monomers (16). The variants of M2-1 were limited to the core domain of the protein. The structure of M2-2 has yet to be resolved; therefore, no domains are assigned to the protein. However, overexpression of M2-2 rearranges the ribonucleocapsid complex, suggesting potential interactions with L (19). Most of the L variants were in the RNA-dependent RNA-polymerase (RdRp), connector domain (CD), and methyltransferase (MT) domains. RdRp is an interaction site for P (37, 38). The complete structures of the CD and MT domains have not yet been determined in the attempts to resolve the full L structure; however, it is suggested that these domains may be dynamic and also interact with the P protein (16, 37, 38). Overall, these associations indicate potential residues of interest in the replication-associated proteins that may impact the fitness of the virus during an infection.

## DISCUSSION

Our in-depth analysis of RSV A sequences shows distinct clusters of variations within replication-associated genes as specific variation(s) in L often associate with variations in one or more of the other replication-associated genes. We grouped these associations, depicted them in a PCA plot, and compared their similarity to relatively known methods of RSV genotyping with the Nextclade tool. The observed associations were not only useful for defining genotypic groups but also supported the advantage of full-length sequence analyses to properly characterize and genotype RSV subgroups. Importantly, our analysis revealed that associations of variants among different replication-associated genes were also present in the related pneumovirus HMPV. These data can be used to infer the dynamic variation of RSV genotypes and their associated pathogenicity in different seasons, as well as a starting point for a detailed investigation of the molecular interactions among proteins from the replication complex.

Most of the reported sequencing analyses from clinical studies generate new sequences based on a known or closely related reference sequence (39–42). An alternative method is *de novo* assembly. The advantage of *de novo* assembly is that it does not require a reference sequence that could introduce bias in the form of variations in the consensus sequence. A merger known as reference-guided *de novo* assembly has also been proposed for larger genome construction (43). In this study, we employed the SPAdes *de novo* assembler with the rnaviralSPAdes option, which is well adapted for shorter reads and metavirome (44, 45). This gives an accurate sequence per isolate as genomic variations including intergenic, or repetitive regions, and indels are also accounted for.

With *de novo* assembly, one must be conscientious when trimming the CDS regions. For RSV, one of the prominent characteristics observed during alignment is the novel duplication in the C-terminal region of the attachment glycoprotein G (25). Of the 109 sequences analyzed in this study, only 10 were without the duplication (GenBank: KY967362, MF001039, PP525326, MN306050, MN306054, PP525323, OK659680, PP525325, PP5253324, MN310477). In addition to the duplication event in the G, we also observed the presence of alternate codons while trimming the CDS regions. We observed that these alternate codons often lead to a few extra or less amino acids that may not be properly annotated during database submissions. Observed alternate codons included seven sequences that have a later stop codon in the G gene (GenBank: OK649680-84, OR287918, OR287948). This alternate codon is also present in the well-studied reference genome—GenBank: KT992094.1. Two sequences were also observed to have a later stop codon in the L gene (GenBank: PP525304, OR143185). To our knowledge these later stop codon has not been previously reported . Other sequences without these later stop codons often have a tandem stop codon e.g., “TAATGA.” These tandem stop codons have been studied in the RSV G gene and shown to decrease the expression of the fusion glycoprotein (F) and facilitate immune evasion of the virus (46, 47). Nine sequences in this study have an alternative start codon in the M2-2 gene (GenBank: PP525314, PP525323-4, OK649682, OK649684, OR143171, OR143187, OR522529, OR601479). M2-2 is generally known to have three start codons, and RSV A2 has this variation too (18, 48). Only the first and second of these start codons have been shown to result in a functional M2-2 protein during replication (18). Alternate start codons in influenza virus have been shown not to affect viral fitness, particularly when the translated protein does not result in a missing domain (49, 50).

Many external and internal factors create the need for viral genomic variation. One unclear question is which protein(s) is/are the major driver of these genomic variations. Pressure or changes from the host immune response can cause adaptations to be selected for in the surface proteins (SH, G, and F). The proteins involved in viral replication (N, P, M2-1, M2-2, and L) may adapt in response to changing levels of replication. The polymerase might alter its speed or fidelity to ensure viral replication fitness. Additionally, with the high error rate of the RdRp, the replication-associated protein functions can play a major role in the variations observed throughout the entire genome. We can also speculate that the drivers of genetic variation may alternate, thereby creating a situation where if one aspect of the virus replication cycle becomes more efficient in its function (e.g., virus entry), a change may be required in a separate function of the virus (e.g., the replication machinery) to maintain a balanced system. This generates a cycle of adaptations making it difficult to pinpoint the originator.

It has been established that the HVR2 of G is highly variable and often used in determining RSV genotypes (51). In this study, we focused on the proteins involved in replication. We observed variations majorly in the L RdRp, CD, and MT domains. Consistent associations were also observed in other replication-associated genes such as the NTD of P, the core domain of M2-1, and M2-2. With similar predictions comparing genotype groups based on the replication-associated genes (Fig. 2A) and genotypes assigned based on the full-length genome (Fig. 2D), we show that genotyping based on the replication-associated genes provides a more consistent and better representation of the entire genomic variation than the genotypes defined by variations in G (Fig. 2E).

As observed in the results, the defining variations are in the interacting domains of the replication-associated proteins. It is known that L, but not P, interacts directly with the RNA template and P interacts with L as a cofactor during replication (52). Established protein prediction also shows that L binds to the C-terminal domain (CTD) of P rather than its NTD (16, 38). However, in the well-studied Vesicular Stomatitis Virus (VSV), it has been shown that the NTD of P interacts with the CTD, RdRp, and CD domains of L (53–55). With such an interactive polymerase complex, it is unsurprising that there are many associated variations between the proteins involved. The variations found in the domains of the P, M2-2, and L proteins may alter their interactions, including the stability of an interaction. This may then impact the functionality of the virus, for example, the speed or fidelity of the polymerase. Improved polymerase function can lead to increased viral replication and pathogenicity. However, further testing will be required to determine how these specific variations impact the replication and pathogenicity of RSV.

An outstanding question is the selection order of these coordinated variations. Unfortunately, in this analysis, we are unable to directly answer this question due to the lack of evolutionary information for these sequences. However, we can speculate the variations are sequentially selected to benefit the virus. One hypothesis for how these coordinated variations occur starts with the polymerase first generating a random variant in a replication-associated gene during replication. As the virus continues to replicate, the function of the variant is revealed. If the variant is detrimental to the virus, it will most likely be removed from the population as it has no benefit. If, however, the variant is neutral, it may remain in the population as it functions normally. In subsequent replication cycles, the polymerase will continue to introduce variations. One of these variations could occur in the interaction site of another replication-associated gene. Now, with the presence of both variants, the interaction between the replication-associated genes is altered. This alteration can have multiple outcomes. However, we hypothesize that in the case of the associated variations observed above, those that have a positive effect on the virus’ function will be selected and remain in the population. A third functional outcome for the first variant generated could be to benefit the virus. If the viral infection is in an environment of low abundance, it may select a variant that improves the polymerase function to increase its replication. As the viral replication increases, the number of mutations also increases. This can generate the additional variants seen in the other replication-associated genes. We speculate these secondary variants may help boost the function of the polymerase or perhaps even neutralize the polymerase function to a balanced state. The observed set of coordinated variations in the viral replication-associated genes may be essential for the virus to maintain its presence in a population.

With the prevalence of circulating RSV among the human population, it is crucial to understand the genomic determinants of RSV pathogenesis. It has been shown that different RSV strains may impact clinical outcomes (47, 56). Yet, there is a lack of consensus on the exact strains that cause the different severities of disease (57). Improving the annotation of variations specifically involved in virus replication may aid in the identification of RSV genotypes that correspond to pathogenesis. The traditional genotyping method based on variations in G is typically preferred in the field due to its lower cost and historical usage. However, genotyping by G lacks valuable information about variations throughout the entire genome. Therefore, we agree with the reccomendation to use whole-genome sequencing as the most representative method for genotyping RSV. While improvements remain to reduce the cost, efficiency, and accessibility for this method, the increased information and diversity discovered using whole-genome sequencing is invaluable. In addition to genotyping using the full RSV sequence, we propose concentrating on the variations in the replication-associated genes as we have shown these are the main determinates of the sequence variations. This method can be implemented in whole-genome analysis to help direct focus on how the various sequences differ or perhaps, furthermore, be incorporated into artificial intelligence tools trained to detect complementary mutations. Moreover, investigating the variation patterns in the CDS of replication-associated genes can be very informative in downstream studies on the efficiency of the polymerase. As we continually search for ways to combat this prevalent virus, these studies can promote a better understanding of how pneumoviruses replicate and their genotype-phenotype correlations.

## MATERIALS AND METHODS

### Description of cohort and data collection

Ninety-six RSV clinical samples were obtained from the Children’s Hospital of Philadelphia (CHOP), Philadelphia, Pennsylvania, USA. Samples used in this study were from 3 different coded cohorts. Cohort H (7 samples; 2012 and 2017), Cohort CL (6 samples; 2013–2015), and Cohort B (83 samples; 2015 and 2016). All clinical samples were nasal washes collected from hospitalized patients between the age of 0 and 2 years as reviewed and approved by the CHOP Institutional Review Board (IRB). From the 96 samples, we could *de novo* assemble full-length sequences for the 31 included in this study. Other sample sequences analyzed outside these cohorts were obtained from the National Center for Biotechnology Information (NCBI Virus) and Bacterial and Viral Bioinformatics Resource Center (BV-BRC) databases (78 sequences; 2012–2023). Complete sequences without insertions in their CDS were selected at random to increase the number of sequences we have each year (Table S2).

### RNA extraction, library preparation, and NGS sequencing

The processing of samples in Cohort H (7 samples) and CL (6 samples) has been previously described in detail (58, 59). For Cohort B (83 samples), total RNA was extracted from clinical samples using TRIzol LS (Invitrogen) according to the manufacturer’s instructions. Linear Acrylamide (Invitrogen) was added at the precipitation step of RNA extraction to increase yield. RNA quantity and quality were measured using Nanodrop and Bioanalyzer (Agilent Technologies).

Sigma SeqPlex RNA Amplification Kit was used for making the complementary deoxyribonucleic acid (cDNA) library preparation for all samples to be sequenced using the Illumina NovaSeq 6000 to generate 150 bp, paired-end reads. The sequencing generated an average of 50 million reads per sample with an average Phred quality score between 34.6 and 36.1.

### *De novo* assembly

The quality of the Illumina paired-end reads was analyzed using FastQC (v0.11), and Illumina adapters were trimmed off the reads using Cutadapt (v2.5) (60). Bowtie2 (v2.4.1) (61) was then used to remove host reads that aligned to the human genome (GRCh38) to generate non-host reads for subsequent analysis. SPAdes (v3.15.5(44) was used for the *de novo* assembly of the non-host paired-end reads of each sample. An additional pipeline option in the assembler (rnaviral) recommended for RNA viral data sets like transcriptomes was included as a parameter while running the assembler with docker on the command line. The contigs and scaffolds generated by the assembler for each sample were aligned against reference sequence—Genbank: KC731482.1 using BLASTN to identify and obtain the RSV A sequences used in this study.

### Variant analysis

MegAlign Pro v17.5.0 (DNASTAR) was used for most of the analysis of the selected sequences of RSV A and HMPV A in this study. The complete nucleotide sequences of all samples were aligned with reference sequences—GenBank: KC731482.1, KT992094.1 for RSV A, and GenBank: NC_039199.1 for HMPV A using MUSCLE with default options. The coding sequences (CDS) of RSV A (NS1, NS2, N, M, P, G, F, SH, M2-1, M2-2, and L) were obtained by trimming the aligned sequences according to the annotation of the references from their feature tables on GenBank. EMBOSS Transeq tool (EMBL-EBI) (62) was used to translate the trimmed CDS to amino acids. The resulting amino acid sequences for each gene were re-uploaded and re-aligned with MUSCLE, and the reference sequences used in trimming the CDS were excluded from subsequent analysis. Variant calling for each CDS was generated using “Compute Variants” in MegAlign Pro v17.5.0 after which these were compiled as reference amino acid (X), reference position (123), variant in the sample (Y), i.e., X123Y. The reference used in this analysis is the consensus sequence generated after the alignment of all the sequences. Statistical analyses for the genetic variation between the CDSs were performed as indicated in the figure legend using GraphPad Prism v.10.

### PCA

PCA graphs were plotted in R Studio v 2023.12.1 + 402 using prcomp function, and PCs were visualized using plotly library. The input data were the uncorrected pairwise distance matrix generated by MegAlign Pro v17.5.0 (DNASTAR) and the demographics of the sequences.

### Phylogenetic tree and clades assignment

Nextstrain and Goya clades were assigned with Nextclade v3.2.0 https://clades.nextstrain.org (36). The phylogenetic trees were predicted using Maximum Likelihood (RAxML) in MegAlign Pro v17.5.0 with the bootstrap iteration of 1000.

## Data Availability

All raw sequencing data used for de novo assembly were deposited in SRA under accession numbers PRJNA837014, PRJNA681672, and PRJNA1108716. All 31 assembled and annotated sequences were submitted in GenBank and were assigned accession numbers PP525(296–326).

## SUPPLEMENTAL MATERIAL

Figure S1 jvi.00990-24-s0001.tif Maximum Likelihood (ML) phylogeny tree of 21 HMPV A full length sequences.

Supplemental material jvi.00990-24-s0002.docx Tables S1, S2, S5, and all supplemental material legends.

Table S3 jvi.00990-24-s0003.xlsx Annotated variations observed in the CDS of replication-associated genes.

Table S4 jvi.00990-24-s0004.xlsx Complete version of Table 4 showing variations observed in more than 2 sequences.

Table S6 jvi.00990-24-s0005.xlsx Table showing assigned Nextstrain clade and Goya clade of each sequence.

Table S7 jvi.00990-24-s0006.xlsx Complete version of Table 5 showing variations that are observed in the CDS of replication-associated genes.

ASM does not own the copyrights to Supplemental Material that may be linked to, or accessed through, an article. The authors have granted ASM a non-exclusive, world-wide license to publish the Supplemental Material files. Please contact the corresponding author directly for reuse.
