# Supplementary figures and images for: Identification of distinct genotypes in circulating RSV A strains based on variants in the virus replication-associated genes
Source: J Virol. 2024 Jul 15;98(8):e00990-24. doi: 10.1128/jvi.00990-24 (PMC11334426; doi:10.1128/jvi.00990-24)

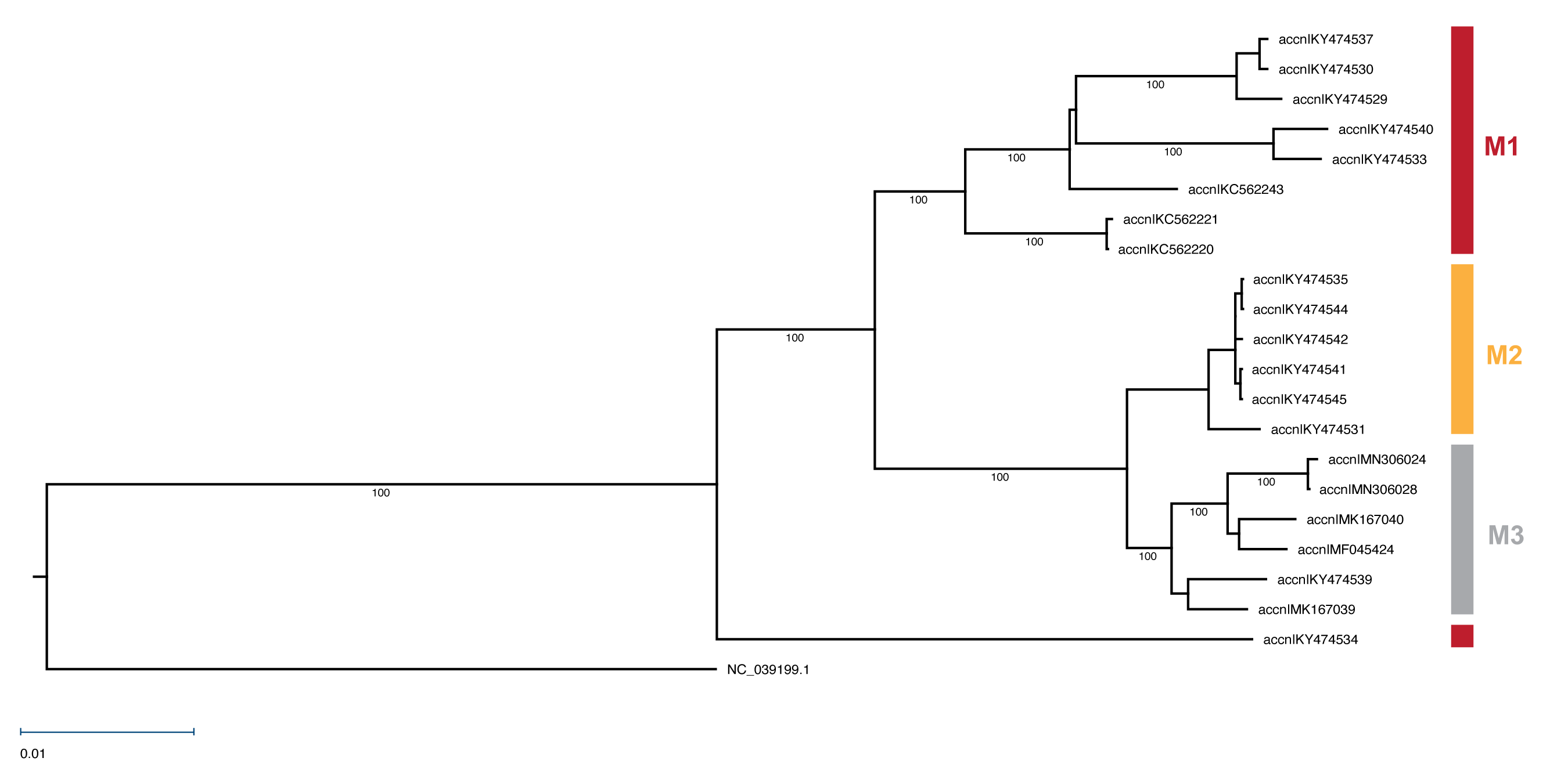

Supplement: Figure S1 — Maximum Likelihood (ML) phylogeny tree of 21 HMPV A full length sequences. [file jvi.00990-24-s0001.tif]
