# Supplementary material for: Identification of distinct genotypes in circulating RSV A strains based on variants in the virus replication-associated genes
Source: J Virol. 2024 Jul 15;98(8):e00990-24. doi: 10.1128/jvi.00990-24 (PMC11334426; doi:10.1128/jvi.00990-24)
Supplement: Supplemental material — Tables S1, S2, S5, and all supplemental material legends. [file jvi.00990-24-s0002.docx]

**SUPPLEMENTARY TABLES AND LEGENDS:**

**Table S1.** Variations in the 31 RSV A sequences from Philadelphia. Computed variations showing the combined number of substitutions, insertions, and deletions per gene. Total variations were deduced from the nucleotide sequence alignment, and non-synonymous variations were deduced from the amino acid alignment.

|  | Total Number of Variations | Number of Non-Synonymous Variations | Non-Synonymous/Total Variations |
| --- | --- | --- | --- |
| NS1 | 60 | 5 | 0.083 |
| NS2 | 45 | 0 | 0.000 |
| N | 120 | 3 | 0.025 |
| P | 58 | 10 | 0.172 |
| M | 81 | 19 | 0.234 |
| SH | 23 | 2 | 0.087 |
| G | 287 | 181 | 0.630 |
| F | 195 | 19 | 0.097 |
| M2-1 | 38 | 6 | 0.158 |
| M2-2 | 30 | 21 | 0.700 |
| L | 709 | 130 | 0.183 |

**Table S2.** GenBank accession number, length of genome, US states, year of sample collection of all 109 RSV A sequences. UNKN indicates sequences of unknown origin within the US. Sequences with asterisks “*” are from samples obtained from the CHOP cohorts.

| Accession Number | Length of genome (bp) | US State | Collection Date |
| --- | --- | --- | --- |
| PP525323* | 15248 | PA | 2012 |
| PP525326* | 15332 | PA | 2012 |
| PP525325* | 15251 | PA | 2012 |
| OK649680 | 15177 | US - UNKN | 2012 |
| OK649681 | 15225 | US - UNKN | 2012 |
| OK649682 | 15242 | US - UNKN | 2012 |
| OR466338 | 15330 | US - UNKN | 2012 |
| OR466339 | 15261 | US - UNKN | 2012 |
| PP525324* | 15017 | PA | 2013 |
| KU950506 | 15202 | US - UNKN | 2013 |
| KX894805 | 15173 | US - UNKN | 2013 |
| KY982516 | 15218 | TN | 2013 |
| MN531557 | 15063 | US - UNKN | 2013 |
| OR466340 | 15258 | US - UNKN | 2013 |
| OR466360 | 15258 | US - UNKN | 2013 |
| OR466361 | 15390 | US - UNKN | 2013 |
| PP525320* | 15060 | PA | 2014 |
| KU839637 | 15173 | TN | 2014 |
| KU950464 | 15232 | US - UNKN | 2014 |
| KU950523 | 15226 | US - UNKN | 2014 |
| KU950537 | 15231 | US - UNKN | 2014 |
| KU950686 | 15231 | US - UNKN | 2014 |
| LC474556 | 15228 | US - UNKN | 2014 |
| LC474557 | 15231 | US - UNKN | 2014 |
| LC474558 | 15231 | US - UNKN | 2014 |
| OK649683 | 15242 | US - UNKN | 2014 |
| PP525298* | 15067 | PA | 2015 |
| PP525299* | 15118 | PA | 2015 |
| PP525296* | 15090 | PA | 2015 |
| PP525309* | 15080 | PA | 2015 |
| PP525310* | 15066 | PA | 2015 |
| PP525297* | 15069 | PA | 2015 |
| PP525319* | 15108 | PA | 2015 |
| KY967362 | 15117 | US - UNKN | 2015 |
| KY967363 | 15203 | US - UNKN | 2015 |
| MF001039 | 14981 | US - UNKN | 2015 |
| OK649684 | 15253 | US - UNKN | 2015 |
| PP525311* | 15062 | PA | 2016 |
| PP525300* | 15070 | PA | 2016 |
| PP525301* | 15111 | PA | 2016 |
| PP525312* | 15070 | PA | 2016 |
| PP525302* | 15021 | PA | 2016 |
| PP525303* | 15063 | PA | 2016 |
| PP525313* | 15075 | PA | 2016 |
| PP525322* | 15179 | PA | 2016 |
| PP525314* | 15112 | PA | 2016 |
| PP525315* | 15056 | PA | 2016 |
| PP525304* | 15167 | PA | 2016 |
| PP525316* | 15077 | PA | 2016 |
| PP525317* | 15127 | PA | 2016 |
| PP525305* | 15100 | PA | 2016 |
| PP525318* | 15107 | PA | 2016 |
| MN630093 | 15178 | AR | 2016 |
| MN630096 | 15141 | AR | 2016 |
| MN630098 | 15102 | AR | 2016 |
| MN630105 | 15067 | AR | 2016 |
| MN630106 | 15160 | AR | 2016 |
| PP525321* | 15330 | PA | 2017 |
| PP525306* | 15252 | PA | 2017 |
| PP525307* | 15361 | PA | 2017 |
| PP525308* | 15258 | PA | 2017 |
| MN306017 | 15260 | US - UNKN | 2018 |
| MN306021 | 15236 | US - UNKN | 2018 |
| MN310477 | 15129 | US - UNKN | 2018 |
| MW033958 | 15267 | TN | 2018 |
| MN306029 | 15191 | US - UNKN | 2019 |
| MN306050 | 15156 | US - UNKN | 2019 |
| MN306054 | 15116 | US - UNKN | 2019 |
| ON729318 | 15233 | US - UNKN | 2019 |
| ON729319 | 15171 | US - UNKN | 2019 |
| OR287841 | 15137 | WA | 2019 |
| OR287842 | 15134 | WA | 2019 |
| OR287846 | 15197 | WA | 2019 |
| OR287849 | 15196 | WA | 2019 |
| OR287859 | 15197 | WA | 2019 |
| OQ331220 | 15264 | WA | 2020 |
| OQ331221 | 15218 | WA | 2020 |
| OR287917 | 15013 | WA | 2020 |
| OR287918 | 15173 | WA | 2020 |
| OR287919 | 15134 | WA | 2020 |
| OR287927 | 15198 | WA | 2020 |
| OR287948 | 15014 | WA | 2020 |
| OR287976 | 15048 | WA | 2020 |
| OR287984 | 15168 | WA | 2020 |
| OR287985 | 15156 | WA | 2020 |
| OP965711 | 15167 | WA | 2021 |
| OR287986 | 15046 | WA | 2021 |
| OR287987 | 15134 | WA | 2021 |
| OR287988 | 15199 | WA | 2021 |
| OP890317 | 15261 | WA | 2022 |
| OP890331 | 15261 | WA | 2022 |
| OP890332 | 15218 | WA | 2022 |
| OQ024110 | 15238 | MA | 2022 |
| OQ024120 | 15255 | MA | 2022 |
| OQ171912 | 15239 | MA | 2022 |
| OQ171931 | 15244 | MA | 2022 |
| OR143176 | 15222 | AZ | 2022 |
| OR143187 | 15224 | AZ | 2022 |
| OR143219 | 15224 | AZ | 2022 |
| OR143160 | 15220 | AZ | 2023 |
| OR143161 | 15162 | AZ | 2023 |
| OR143163 | 15221 | AZ | 2023 |
| OR143171 | 15224 | AZ | 2023 |
| OR143184 | 15226 | AZ | 2023 |
| OR143185 | 15231 | AZ | 2023 |
| OR522508 | 15172 | OR | 2023 |
| OR522529 | 15197 | OR | 2023 |
| OR601479 | 15197 | WA | 2023 |
| OR601480 | 15197 | OR | 2023 |

**Table S3:** Annotated variations observed in the CDS of replication-associated genes (N, P, M2, L) of selected 109 RSV A sequences. Sequences with asterisks “*” are from samples obtained from the CHOP cohorts. An asterisk “*” in the annotation indicates that the sequence has no variation in the gene when compared to the consensus sequence. **See Excel file uploaded**.

**Table S4:** Complete version of Table 4 showing variations observed in more than 2 sequences. Sequences with asterisks “*” are from samples obtained from the CHOP cohorts. Variations are arranged by their positions in the CDS and assigned to one of the groups R1-R6. **See Excel file uploaded**.

**Table S5:** Distribution of 109 sequences within each predicted groups including the year of sample collection and their locations in the US by states. UNKNs indicate that sequences are of unknown origin within the US.

| **Group Name** | **Number of Sequences** | **Range of Years** | **US Locations** |
| --- | --- | --- | --- |
| **R1** | 43 | 2012-2020 | PA, TN, WA, UNKNs |
| **R2** | 17 | 2021-2023 | AZ, WA, UNKNs |
| **R3** | 32 | 2015-2023 | AR, AZ, MA, OR, TN, WA, UNKNs |
| **R4** | 12 | 2016-2021 | PA, UNKNs |
| **R5** | 3 | 2019 | UNKNs |
| **R6** | 2 | 2012, 2014 | OR, UNKNs |

**Table S6:** Table showing assigned Nextstrain clade and Goya clade of each sequence compared to our predicted R1-R6 groups. Sequences with asterisks “*” are from samples we obtained from the CHOP cohorts. Clades were determined using full-length sequences in the Nextclade tool. **See Excel file uploaded**.

**Table S7:** Complete version of Table 5 showing variations that are observed in the CDS of replication-associated genes (N, P, M2, L) of the selected 21 HMPV A sequences. Variations included are observed in more than 2 sequences. An asterisk “*” indicates that the sequence has no variation in the gene when compared to the consensus sequence. **See Excel file uploaded**.

**Figure S1:** Maximum Likelihood (ML) phylogeny tree of 21 HMPV A full length sequences. Color bars represents assigned groups based on the variation patterns observed in the replication-associated genes. The tree was rooted on the reference sequence - GenBank: NC_039199.1.
